# Efficacy and Safety of Evenamide, a glutamate modulator: Results from a Phase 2/3, international, randomized, double-blind, placebo-controlled add-on trial

**DOI:** 10.1192/j.eurpsy.2025.706

**Published:** 2025-08-26

**Authors:** R. Anand, A. Turolla, G. Chinellato, R. Giuliani, F. Sansi, R. Hartman

**Affiliations:** 1 R&D, APC, St Moritz, Switzerland; 2 R&D, Newron Pharmaceuticals SpA, Milan, Italy; 3 R&D, NeurWrite LLC, Morristown, United States

## Abstract

**Introduction:**

Treatment of schizophrenia remains a challenge despite the development of numerous antipsychotics (AP) (Pompili et al CNS NDDT 2017; 16 870-884). The various strategies to address patients’ unmet needs, such as AP polypharmacy or switching AP, do not seem to provide adequate benefit. A reason for this may reside in the similarity of mechanisms of action of most APs. However, the monoaminergic systems targeted by these drugs are not always disrupted in schizophrenia, as it is widely accepted that patients who fail to respond to AP have glutamatergic, but not dopaminergic dysfunctions (Moghaddam et al NPP 2012; 37 4-15). Evenamide normalizes excessive glutamate release without affecting its basal levels, and it does not interact with >150 CNS targets. It has been shown to be effective in animal models of psychosis as monotherapy and add-on to AP. Previous trials indicate that evenamide benefits patients with schizophrenia with inadequate response and have shown clinically important benefits lasting up to 1-year in TRS (Anand et al IJNPP 2023; 26 523-528).

**Objectives:**

Study 008A, a phase 2/3 international, randomized, double-blind, placebo-controlled, 4-week trial, assessed the efficacy and safety of evenamide 30 mg *bid* as add-on in patients poorly responding to an SGA.

**Methods:**

Outpatients with a diagnosis of schizophrenia, still symptomatic (PANSS 70-85; CGI-S 4-6) despite treatment with an SGA at a therapeutic dose (confirmed through plasma levels) for an adequate period, were enrolled. Efficacy was assessed on the PANSS, CGI-S, and LOF through the comparison of changes from baseline to Day 29 between evenamide and placebo groups using a MMRM analysis. Moreover, the proportion of patients reaching a clinically important improvement on the PANSS and CGI-C were compared between groups using a logistic regression model (chi-square test). Safety measures comprised: TEAEs, vital signs, labs, ECG/EEG, seizure checklist, physical/neurological/eye examinations, C-SSRS, ESRS-A, CDSS.

**Results:**

291 patients were randomized in the study in 11 countries (EU, Asia and LATAM), and 280 completed the study. The low attrition rate (<4%), and proportion of patients with TEAEs (~25% in both groups) indicate that evenamide was well tolerated. A statistically significant greater improvement was found on the PANSS total score and on the CGI-S in the evenamide compared to the placebo group. Furthermore, a significantly higher proportion of patients treated with evenamide, compared to those receiving placebo, experienced clinically meaningful benefit measured on the PANSS (≥20% improvement) and CGI-C (at least much improved).

**Conclusions:**

This is the first randomized, placebo-controlled trial to demonstrate the clinical benefit of adding a NCE modulating glutamate in patients with schizophrenia who did not experience adequate response from treatment with a SGA.

**Disclosure of Interest:**

R. Anand Consultant of: AbbVie, Acadia, BiolineRx, Domain, Enkam, Erydel, Forest, Janssen, Hoffman La Roche, Lundbeck, Noema, Ono, Pfizer, UCB, Shire, Sigma-Tau, Takeda, Teva, A. Turolla Employee of: Newron Pharmaceuticals SpA, G. Chinellato Employee of: Newron Pharmaceuticals SpA, R. Giuliani Employee of: Newron Pharmaceuticals SpA, F. Sansi Employee of: Newron Pharmaceuticals SpA, R. Hartman Consultant of: Newron Pharmaceuticals SpA.

